# Development of a food frequency questionnaire to estimate habitual dietary intake in Japanese children

**DOI:** 10.1186/1475-2891-9-17

**Published:** 2010-04-10

**Authors:** Tomomi Kobayashi, Sanae Tanaka, Chihiro Toji, Hideko Shinohara, Miharu Kamimura, Naoko Okamoto, Shino Imai, Mitsuru Fukui, Chigusa Date

**Affiliations:** 1 Department of Food and Nutritional Sciences, School of Natural Science and Ecological Awareness, Graduate School of Humanities and Sciences, Nara Women's University, Kitauoya-nishimachi, Nara, 630-8506, Japan; 2 Department of Food Science and Nutrition, Human Environmental Sciences, Mukogawa Women's University, 6-46 Ikebiraki-cho, Nishinomiya, Hyogo 663-8558, Japan; 3 Department of Food Sciences and Nutrition, Faculty of Human Life and Environment, Nara Women's University, Kitauoya-nishimachi, Nara, 630-8506, Japan; 4 Laboratory of Statistics, School of Medicine, Osaka City University, 1-4-3 Asahi-machi, Abeno-ku, Osaka, 545-8585, Japan

## Abstract

**Background:**

Food frequency questionnaires (FFQ) are used for epidemiological studies. Because of the wide variations in dietary habits within different populations, a FFQ must be developed to suit the specific group. To date, no FFQ has been developed for Japanese children. In this study, we developed a FFQ to assess the regular dietary intake of Japanese children. The FFQ included questions regarding both individual food items and mixed dishes.

**Methods:**

Children (3-11 years of age, n = 621) were recruited as subjects. Their parents or guardians completed a weighed dietary record (WDR) for each subject in one day. We defined FOOD to be not only as a single food item but also as a mixed dish. The dieticians conceptually grouped similar FOODs as FOOD types. We used a contribution analysis and a multiple regression analysis to select FOOD types.

**Results:**

We obtained a total of 586 children's dietary data (297 boys and 289 girls). In addition, we obtained 1,043 FOODs. Dieticians grouped into similar FOODs, yielding 275 FOOD types. A total of 115 FOOD types were chosen using a contribution analysis and a multiple regression analysis, then we excluded overlapping items. FOOD types that were eaten by fewer than 15 subjects were excluded; 74 FOOD types remained. We also added liver-based dishes that provided a high amount of retinol. A total of 75 FOOD types were finally determined for the FFQ. The frequency response formats were classified into four type categories: seven, eight, nine and eleven, according to the general intake frequency of each FOOD type. Information on portion size was obtained from the photographs of each listed FOOD type in real scale size, which was the average amount of the children's portion sizes.

**Conclusions:**

Using both a contribution analysis and a multiple regression analysis, we developed a 75-food item questionnaire from the study involving 586 children. The next step will involve the verification of FFQ reproducibility and validity.

## Background

Long-term eating habits affect lifestyle-related diseases including obesity, diabetes, and cardiovascular diseases. In recent years, obesity has become prevalent not only among adults but also in children in Japan [1,2]. Those who are obese in childhood tend to remain obese as adults [3-6]. When children are overweight, they are more likely to develop metabolic syndrome later in life [7]. Furthermore, the longer an individual is overweight, the greater their risk of cardiovascular disease [8]. Various factors contribute to obesity, including physical inactivity, an irregular and unbalanced diet, and over-eating [9]. Dietary habits are formed during childhood [10]. To prevent adult obesity, it is desirable that individuals acquire appropriate dietary habits in childhood. Habitual dietary intake among children should be assessed to evaluate childhood dietary problems, enabling the correction of any bad dietary habits.

In general, a food frequency questionnaire (FFQ) is used in large-scale epidemiological studies. FFQs are often used because they are more cost-effective and easier to administer than weighed dietary records (WDR) or 24-hour recall [11]. There have been a number of reports using FFQs to assess dietary intake in adults [12,13]. A FFQ originally developed for adults is applied to the assessment of dietary intake in children; however, it has been reported that the children's intake is overestimated when assessed using the FFQ for adults [14-17]. Since it is necessary to develop specific FFQs for the group being studied (to accurately reflect socioeconomic, cultural, and seasonal differences), it would therefore be useful to develop a specific FFQ for children [18]. However, to date no FFQ has been developed and tested in Japanese children [12].

In Japan, some FFQs incorporate raw single food or food groups, such as meat, fish, egg and vegetables [12]. However, a typical Japanese meal normally consists of steamed rice, soup, main dishes, and multiple side dishes. These dishes include various food items and seasonings, and are served as chopped foods that are mixed and/or cooked. This meal format is similar in other Asian countries. Given the mixed nature of these meals, it is difficult for Asian subjects to estimate their intake of single ingredients in the meals prior to cooking, especially if the subjects did not prepare or cook their own meals [19,20]. In order to enable the subjects to answer a FFQ more accurately, a questionnaire as presented below must be developed. The FFQ includes questions about both individual food items and mixed dishes. Therefore, we developed a FFQ specifically for Japanese children, which included questions about both individual food items and mixed dishes based upon the typical eating habits of normal Japanese children.

## Methods

### Subjects

All children (3-11 years of age, n = 621) who were registered at the kindergarten or elementary school attached to Nara Women's University were included as subjects. The study aim and methods were explained orally, and written information was provided to all parents/guardians (i.e., the person who prepared the child's diet). Then, the parents/guardians provided their written informed consent. The study was approved by the research ethics committee of the Faculty of Human Life and Environment of Nara Women's University.

### Weighed dietary record for developing a food list

The parents/their guardians completed a weighed dietary record (WDR) for each subject on one day between May and July 2007. Parents/guardians freely decided the investigation day from among the schedule, which was preliminarily allocated for 3-4 days. A digital cooking scale (TANITA digital cooking scale; KD-402-WH, Tokyo, Japan) was used to weigh all foods. The parents/guardians took two photos of each food item on the digital cooking scale and its weight before and after cooking. After the investigation day, the parents/guardians submitted a CD of the photographic data, with a form detailing each menu according to breakfast, lunch, dinner, or snack on the investigation day, to us.

Registered dieticians determined the child's daily intake of food items by looking at the submitted photographs. In Japan, the approximate sodium percent of all the ingredients except seasonings for each dish were decided. The dieticians used this percentage and estimated the levels of sodium intake.

We then calculated the nutrient intake for each child using the Standard Tables of Food Composition in Japan (fifth revised and enlarged edition) [21].

### FOODs and FOOD types

We defined FOOD as not only a single food item (e.g., apple) but also as a mixed dish (e.g., chirashi-zushi (garnished sushi)). We calculated the composition of nutrients in the FOOD per 100 g. FOODs were then consolidated into mutually exclusive FOOD types, according to form, type of preparation, and nutrient density by the registered dieticians.

### Statistical analyses

We used two methods to develop the list of FOOD types. First, we used the method reported by Block et al. [22] and ranked all of the reported FOOD types according to the contribution analysis. The ranking was based on the percentage of nutrients that each FOOD type contributed to the total nutrient intake of all the subjects. We were especially interested in the total energy, protein, fat, carbohydrate, sodium, and retinol intakes. The percentages were calculated by dividing the nutrient contents of each FOOD type by the total nutrient amounts. All of the FOOD types that contributed at least 0.15% to the total energy and nutrients were combined. Next, multiple regression analysis was conducted in order to list FOOD types identified based on the between-person variance in the intake of specific nutrients. We carried out a multiple regression analysis with the dependent variable as the average energy intake and nutrient intakes of protein, fat, and sodium, and the independent variable as the intake of FOOD types. We selected the FOOD types with up to a 0.90 cumulative square of the multiple correlation coefficients (R^2^). We then combined the selected FOOD types using these two methods and excluded overlapping items. In addition, we excluded FOOD types eaten by fewer than 15 subjects.

We used the statistical software package SPSS for Windows 17.0 (SPSS Inc., Tokyo, Japan) to perform the statistical analyses.

## Results

### Subjects

The parents/guardians of 592 subjects out of the total 621 provided informed consent. We obtained 586 (297 boys and 289 girls) subjects' dietary data. The survey was completed by their parents/guardians. There were 134 children in kindergarten (3-5 years of age), 232 in the lower grades of elementary school (6-8 years of age), and 220 in the upper grades of elementary school (9-11 years of age). The response proportion was 94.3%. In all, 60.4, 23.4, and 16.3% of the total parents/guardians chose to record their food intake for the survey on the weekdays, Saturday, and Sunday, respectively.

### Development of a food list

We obtained 1,043 FOODs in this study. The dieticians conceptually grouped similar FOODs, yielding 275 FOOD types. We performed a contribution analysis. Then, after excluding overlapping items, we obtained 162 FOOD types. We next conducted a step-wise multiple regression analysis. After the analysis, the numbers of selected items for energy, protein, fat, and sodium were 125 (R^2 ^= 0.89), 94 (R^2 ^= 0.81), 55 (R^2 ^= 0.65), and 60 (R^2 ^= 0.59), respectively. The top 10 FOOD types ranked according to R^2 ^values are listed in Table 1. Steamed rice was the highest for energy and protein. Milk and miso soup were ranked at the top for fat and sodium, respectively. We excluded overlapping items and obtained 162 FOOD types.

**Table 1:** FOOD types most predictive of between-person variations in energy, protein, fat, and sodium

Nutrients	Rank	FOOD types	**Cumulative R**^2^*
Energy	1	Steamed white rice	0.06
	2	Milk	0.11
	3	Japanese green tea	0.15
	4	Steamed rice with mixed ingredients	0.19
	5	Cake	0.22
	6	Ice cream	0.25
	7	Rice balls	0.28
	8	Cold noodle	0.31
	9	Cocoa	0.33
	10	Potato salad	0.35
Protein	1	Steamed white rice	0.07
	2	Steamed rice with mixed ingredients	0.13
	3	Milk	0.18
	4	Japanese green tea	0.21
	5	Sashimi (raw fish or raw shellfish)	0.24
	6	Cold noodles	0.27
	7	Broiled fish (dark meat fish)	0.30
	8	Sunny-side up or ham and eggs	0.32
	9	Rice balls	0.34
	10	Broiled fish (lean meat fish)	0.36
Fat	1	Milk	0.05
	2	Potato salad	0.09
	3	Ice cream	0.12
	4	Vegetable salad	0.15
	5	Cake	0.18
	6	Sausage	0.21
	7	Cocoa	0.23
	8	Japanese green tea	0.26
	9	Snacks	0.27
	10	Fried meat	0.29
Sodium	1	Miso soup	0.05
	2	Rice balls	0.09
	3	Soups	0.12
	4	Scrambled eggs	0.16
	5	Steamed rice with mixed ingredients	0.18
	6	Vegetable salad	0.21
	7	Wheat noodle	0.23
	8	Hamburger steak	0.25
	9	Sunny-side up or ham and eggs	0.27
	10	Semi-dried sardine fries	0.29

*:Data are based on cumulative R^2 ^values in a stepwise regression analysis of a food frequency questionnaire.

We then combined the selected FOOD items using the two methods and excluded overlapping items, and we finally obtained 115 FOOD types. In addition, the FOOD types eaten by fewer than 15 subjects were excluded; 74 FOOD types remained. A total of 75 FOOD types were present after the inclusion of a liver-based dish that provides a high amount of retinol. Two additional FOOD types as indicated by the multiple regression analysis, toasted purple laver and boiled edamame (immature soybeans), were added to the existing FOOD types selected using a contribution analysis. The selected FOOD types are shown in Table 2. The question items included staple foods (12 items), main dishes (25), side dishes (15), beverages (3), dairy products (6), sweets and baked goods (9), and fruits (5).

**Table 2:** Complete food list included in the Food Frequency Questionnaire

Staple foods	
1	Steamed white rice or rice with mixed ingredients
2	Rice balls
3	Curried rice or fried rice
4	Donburi (rice topped with chicken and egg or rice topped with beef and egg)
5	Sushi
6	White bread
7	Hot dog
8	Cooked bread, danish pastry bread, or a sweet roll
9	Roll bread and raisin bread
10	Noodle
11	Fried Chinese noodle or spaghetti
12	Okonomiyaki (Japanese pizza with vegetables or pizza)
	
Main dishes	
13	Roasted meat
14	Fried meat
15	Meat and vegetable stew
16	Meat balls
17	Syumai (Chinese steam meat dumpling) or spring roll
18	Gyoza (Chinese meat dumpling)
19	Ham
20	Sausage or bacon
21	Meat roll
22	Croquette
23	Liver-based dishes
24	Sashimi (raw fish or raw shellfish)
25	Broiled fish (white meat fish or shrimp)
26	Broiled fish (lean meat fish or dark meat fish)
27	Stewed fish
28	Fried fish
29	Vinegar-flavored seafood and vegetables
30	Kamaboko (fish paste)
31	Semi-dried sardine fries
32	Fried egg roll
33	Soft-boiled egg
34	Tamago-dofu (steamed beaten egg with soup stock)
35	Cold tofu
36	Natoo (fermented soy beans)
37	Tofu dishes
	
Side dishes	
38	Vegetable salad
39	Tomato
40	Fried meat (with vegetables)
41	Fried vegetables or potatoes
42	Sauteed thin sliced burdock root
43	Boiled vegetables
44	Stewed vegetables or stewed potatoes
45	Boiled or marinated vegetables
46	Fried vegetables or fried potatoes
47	Boiled edamame (immature soybeans)
48	Salt-flavored pickled vegetables
49	Stewed hijiki (sea vegetable)
50	Toasted purple laver
51	Soup
52	Potage or stew
	
Beverages	
53	Japanese green tea
54	Vegetable juice or fruit juice
55	Cocoa or tea or coffee
	
Dairy products	
56	Whole milk
57	Low fat milk or milk coffee
58	Lactic acid bacteria beverage
59	Yogurt
60	Ice cream
61	Cheese
	
Sweets and baked goods	
62	Cake (including doughnuts)
63	Snacks
64	Jelly
65	Biscuit
66	Chocolate
67	Candy
68	Japanese rice cracker
69	Japanese traditional confectionery
70	Pudding
	
Fruits	
71	Banana
72	Kiwi fruits
73	Citrus fruits
74	Apple
75	Strawberry

The Standard Tables of Food Composition in Japan include energy and 40 nutrients. Table 3 shows the cumulative contribution proportion of food to total energy, and the 40 nutrients involved in food intake are present in the newly developed FFQ. The 75 FOOD types had an average of 94.9% coverage energy and nutrient intake. The maximum and minimum nutrient values were 97.8% (Alpha-carotene) and 88.6% (Vitamin C), respectively.

**Table 3:** Percentage of energy and 40 nutrients^† ^involved in food intake in the developed FFQ

Nutrient	Contribution*(%)
Energy (kcal)	95.5
Protein (g)	95.5
Fat (g)	95.7
Carbohydrates (g)	95.1
Sodium (mg)	95.9
Potassium (mg)	93.3
Calcium (mg)	94.7
Magnesium (mg)	94.5
Phosphorous (mg)	95.5
Iron (mg)	94.9
Zinc (mg)	94.4
Copper (mg)	94.9
Manganese (mg)	91.2
Retinol (μg)	97.4
Alpha-carotene (μg)	97.8
Beta-carotene (μg)	95.7
Cryptoxanthin (μg)	93.4
Beta-carotene equivalent (μg)	95.7
Retinol activity equivalent (μg RAE)	96.5
Vitamin D (μg)	92.5
Alpha-tocopherol (mg)	95.0
Beta-tocopherol (mg)	95.7
Gamma-tocopherol (mg)	95.2
Delta-tocopherol (mg)	94.4
Vitamin K (mg)	97.2
Vitamins B1 (mg)	95.1
Vitamins B2 (mg)	95.6
Niacin (mg)	94.5
Vitamin B6 (mg)	94.5
Vitamin B12 (μg)	95.8
Folic acid (μg)	92.2
Pantothenic acid (mg)	95.8
Vitamin C (mg)	88.6
Saturated fatty acid (g)	95.6
Monounsaturated fatty acid (g)	96.1
Polyunsaturated fatty acid (g)	95.5
Cholesterol (mg)	96.7
Soluble dietary fiber (g)	93.5
Insoluble dietary fiber (g)	93.7
Total dietary fiber (g)	93.5
Sodium chloride equivalent (mg)	96.0

† The standard tables of food composition in Japan include energy and 40 nutrients.

* The proportion that the 75 food items occupy in the total intake of energy and 40 nutrients of the 586 subjects.

### Frequency response formats

We considered that the dietary intake of growing children is likely to change over a short period. The FFQ included frequency response formats to recall each child's diet during the past month. The FOOD type intake frequencies were classified into four types: seven (i.e., everyday, 5-6 times per week, 3-4 times per week, 1-2 times per week, 2-3 times per month, 1 time per month, or never), eight ("2-3 times per day" was added to seven categories), nine ("4-5 times per day" was added to eight categories) and eleven ("8-10 times per day", "6-7 times per day" were added to nine categories) according to general intake frequency of each FOOD type. For example, Japanese people drink the listed beverages, especially Japanese green tea, more than once per day. Therefore, we included eleven categories in the beverage and milk category.

### Portion size

We used the photographs of each listed FOOD type to estimate one portion. When the FOOD types were mixed dishes, the median of the amounts eaten by the children was adopted as the standard portion size. When the FOOD types were single food items, we adopted the mean value of the intake of the children who ate the food as the standard portion size.

To determine the intake of steamed rice as accurately as possible, three sizes of rice bowls (large, medium, and small) were included in the questionnaire. The estimation of portion size was classified into six categories referring to the photographs in full-scale size; that is, one-third, one-half, the same amount, 1.5 times, twice, and 'other'.

### School lunch

In Japan, children are provided with lunch in elementary school on weekdays. All elementary students eat the same dishes in their school lunch. We added a question to the developed FFQ concerning the amount and frequency of school lunch intake for elementary school children. The approximate amount of one serving for children offered in the school lunch is decided according to age. We asked the number of times each child ate a school lunch during the past month in the question. We obtained the menu provided by the school during the previous month, and classified the dishes into eight items: rice dishes, breads, noodles, meat dishes, fish dishes, vegetable dishes, desserts, and dairy products. In this question, we inquired about the amount of each food serving eaten at one sitting.

## Discussion

A general WDR is quantified either by weighing or determining volumes using a household measuring tool, such as standard measuring cups and spoons, and a ruler for measuring dimensions. Usually, general WDR performers weigh the raw ingredients [23]. Our WDR was designed to make it easier to assess food portions and composition because we used a digital cooking scale as an index of the size of the dish. We confirmed that the Spearman's correlation coefficient between the general WDR and our WDR were 0.88, 0.81, 0.65, 0.88, and 0.50 for energy, protein, fat, carbohydrate, and sodium intake, respectively, in the preliminary investigation. In the Japanese diet, seasonings such as soy sauce are important dietary elements; however, it is difficult to estimate the intake of these foods from dietary photographs alone. Our WDR was able to give a moderate estimate of sodium intake. To obtain the necessary open-ended data from children, we conducted our WDR instead of using a general WDR. The open-ended data generated by our WDR identified the FOOD types that contribute most importantly to the total absolute intake of a nutrient by the group as a whole. An advantage of this open-ended approach is that important contributors to nutrient intake are unlikely to be missed. Many arbitrary decisions, however, must be made regarding the collapsing of variables, as the open-ended methods are typically coded in much finer detail than would be appropriate for items on a questionnaire [11]. Therefore, we combined the multiple regression analysis to identify the FOOD types based on between-person variance with the contribution analysis. Finally, with two methods we obtained a list of 75 food items that are most important within the diets of Japanese children.

Willett reported the limit for the number of food items as 130 [11]. This is because the burden on subjects to respond to questions increases when there are many food items. Date et al. developed a FFQ from the basic dietary data of 805 Japanese adults, and adopted 122 items [19]. Another study by Tokudome et al. developed a FFQ from 351 Japanese adults, and included 102 items [24]. Cade et al. reported the distribution of several food items in which FFQs were used from 227 studies in a review, and reported that the median number of food items was 79 (range, 5-350 items) [13]. The number of food items in our FFQ is 75, which we consider to be the optimal number of items that can provide an accurate picture of dietary habits without becoming a burden to the study participants.

There were, however, two limitations associated with this study. First, our FFQ does not encompass the seasonal variation in the 75 food items, because the time when we conducted WDR for developing the food list was only one season. However, there are different fruits available in different seasons in Japan. To address this, we decided to add seasonal fruits for future investigation. In addition, because the subjects' parents/guardians freely decided the day for the WRD, it was possible that a special day (e.g. a holiday or other celebration where special foods are eaten) may have been selected. Therefore, when we explained the study method to the parents/guardians, we asked them to eat habitual diets on the day they selected for the WRD.

Finally, the coverage rates of energy and all nutrients were more than 90% except for Vitamin C. Therefore, the FFQ developed for children is considered to be a useful modality for estimating the intake of nutrients.

## Conclusions

Using both a contribution analysis and a multiple regression analysis, we developed a 75-food item questionnaire from the study involving 586 children. In future, we will verify the reproducibility and validity of this FFQ.

## Competing interests

The authors declare that they have no competing interests.

## Authors' contributions

TK conceptualized the design of the study and study protocol, as well as performed data collection, statistical analyses and writing of the manuscript. ST, CT, HS, MK NO and SI contributed to the data collection. MF performed statistical analyses. CD contributed to the concept and design of the study and study protocol, as well as performed data collection and assisted in writing and editing the manuscript. All authors read and approved the final manuscript.

## References

[B1] Kotani K Nishida M Yamashita S Funahashi T Fujioka S Tokunaga K Ishikawa K Tarui S Matsuzawa Y Two decades of annual medical examinations in Japanese obese children: do obese children grow into obese adults? Int J Obes Relat Metab Disord 1997 21 912 921 10.1038/sj.ijo.0800492 9347410

[B2] Kouda K Nakamura H Tokunaga R Takeuchi H Trends in levels of cholesterol in Japanese children from 1993 through 2001 J Epidemiol 2004 14 78 82 10.2188/jea.14.78 15242063 PMC8685174

[B3] Freedman DS Khan LK Serdula MK Dietz WH Srinivasan SR Berenson GS The relation of childhood BMI to adult adiposity: the Bogalusa Heart Study Pediatrics 2005 115 22 27 15629977 10.1542/peds.2004-0220

[B4] Guo SS Huang C Maynard LM Demerath E Towne B Chumlea WC Siervogel RM Body mass index during childhood, adolescence and young adulthood in relation to adult overweight and adiposity: the Fels Longitudinal Study Int J Obes Relat Metab Disord 2000 24 1628 1635 10.1038/sj.ijo.0801461 11126216

[B5] Field AE Cook NR Gillman MW Weight status in childhood as a predictor of becoming overweight or hypertensive in early adulthood Obes Res 2005 13 163 169 10.1038/oby.2005.21 15761176 PMC1989681

[B6] Whitlock EP Williams SB Gold R Smith PR Shipman SA Screening and interventions for childhood overweight: a summary of evidence for the US Preventive Services Task Force Pediatrics 2005 116 e125 e144 10.1542/peds.2005-0242 15995013

[B7] Vanhala M Vanhala P Kumpusalo E Halonen P Takala J Relation between obesity from childhood to adulthood and the metabolic syndrome: population based study BMJ 1998 317 319 9685277 10.1136/bmj.317.7154.319 PMC28624

[B8] Baker JL Olsen LW Sørensen TI Children body-mass index and the risk of coronary heart disease in adulthood N Engl J Med 2007 357 2329 2337 10.1056/NEJMoa072515 18057335 PMC3062903

[B9] Sugiura R Sakamoto M Murata M Risk of life-style related diseases in young children Jpn J Nutri Diete 2007 65 67 73 (in Japanese)

[B10] Mikkilä V Räsänen L Raitakari OT Pietinen P Viikari J Consistent dietary patterns identified from childhood to adulthood: the cardiovascular risk in Young Finns Study Br J Nutr 2005 93 923 931 10.1079/BJN20051418 16022763

[B11] Willett W Willett W Food-frequency methods Nutritional epidemiology 1998 2 Oxford: Oxford University Press 74 100 full_text

[B12] Wakai K A review of food frequency questionnaires developed and validated in Japan J Epidemiol 2009 19 1 11 10.2188/jea.JE20081007 19164867 PMC3924089

[B13] Cade J Thompson R Burley V Warm D Development, validation and utilisation of food-frequency questionnaires - a review Public Health Nutr 2002 5 567 587 10.1079/PHN2001318 12186666

[B14] Wilson AM Lewis RD Disagreement of energy and macronutrient intakes estimated from a food frequency questionnaire and 3-day diet record in girls 4 to 9 years of age J Am Diet Assoc 2004 104 373 378 10.1016/j.jada.2003.12.021 14993859

[B15] Fumagalli F Pontes Monteiro J Sartorelli DS Vieira MN de Lourdes M Pires Bianchi Validation of a food frequency questionnaire for assessing dietary nutrients in Brazilian children 5 to 10 years of age Nutrition 2008 24 427 432 10.1016/j.nut.2008.01.008 18343639

[B16] Serdula MK Alexander MP Scanlon KS Bowman BA What are preschool children eating? A review of dietary assessment Annu Rev Nutr 2001 21 475 498 10.1146/annurev.nutr.21.1.475 11375446

[B17] Bertoli S Petroni ML Pagliato E Mora S Weber G Chiumello G Testolin G Validation of food frequency questionnaire for assessing dietary macronutrients and calcium intake in Italian children and adolescents J Pediatr Gastroenterol Nutr 2005 40 555 560 10.1097/01.MPG.0000153004.53610.0E 15861015

[B18] Kristal AR Feng Z Coates RJ Oberman A George V Associations of race/ethnicity, education, and dietary intervention with the validity and reliability of a food frequency questionnaire: the Women's Health Trial Feasibility Study in Minority Populations Am J Epidemiol 1997 146 856 869 9384206 10.1093/oxfordjournals.aje.a009203

[B19] Date C Yamaguchi M Tanaka H Development of a food frequency questionnaire in Japan J Epidemiol 1997 6 131S 136S 10.2188/jea.6.3sup_131 8800285

[B20] Ahn Y Kwon E Shim JE Park MK Joo Y Kimm K Park C Kim DH Validation and reproducibility of food frequency questionnaire for Korean genome epidemiologic study Eur J Clin Nutr 2007 61 1435 1441 10.1038/sj.ejcn.1602657 17299477

[B21] Ministry of Education Culture Sports, Science and Technology Standard tables of food composition in Japan fifth revised and enlarged edition 2005 Tokyo: National Printing Bureau (in Japanese)

[B22] Block G Hartman AM Dresser CM Carroll MD Gannon J Gardner L A data-based approach to diet questionnaire design and testing Am J Epidemiol 1986 124 453 469 3740045 10.1093/oxfordjournals.aje.a114416

[B23] Buzzard Marilyn Willett W 24-hour dietary recall and food record methods Nutritional epidemiology 1998 2 Oxford: Oxford University Press 50 73

[B24] Tokudome S Ikeda M Tokudome Y Imaeda N Kitagawa I Fujiwara N Development of data-based semi-quantitative food frequency questionnaire for dietary studies in middle-aged Japanese Jpn J Clin Oncol 1998 28 679 687 10.1093/jjco/28.11.679 9861235

